# Single View Techniques for Modelling Coronary Pressures Losses. Comment on Tsigkas et al. Rapid and Precise Computation of Fractional Flow Reserve from Routine Two-Dimensional Coronary Angiograms Based on Fluid Mechanics: The Pilot FFR2D Study. *J. Clin. Med.* 2024, *13*, 3831

**DOI:** 10.3390/jcm14061958

**Published:** 2025-03-14

**Authors:** Daniel J. Taylor, Tom Newman, Julian Gunn, Paul D. Morris

**Affiliations:** 1 Division of Clinical Medicine, School of Medicine & Population Health, University of Sheffield, Sheffield S10 2RX, UK; t.a.newman@sheffield.ac.uk (T.N.); j.gunn@sheffield.ac.uk (J.G.); paul.morris@sheffield.ac.uk (P.D.M.); 2 Insigneo Institute for in silico Medicine, Sheffield S10 2RX, UK; 3 Biomedical Research Centre, Sheffield Teaching Hospitals NHS Foundation Trust, Sheffield S10 2JF, UK; 4 Department of Cardiology, Sheffield Teaching Hospitals NHS Foundation Trust, Sheffield S5 7AU, UK

We have read the research article “Rapid and Precise Computation of Fractional Flow Reserve from Routine Two-Dimensional Coronary Angiograms Based on Fluid Mechanics: The Pilot FFR2D Study” by Tsigkas et al. [1] with interest.

The authors described a computational model for deriving virtual fractional flow reserve (vFFR) from a single 2D coronary angiographic image, which was validated against invasive measurements. They concluded that their technique provided comparable accuracy with 3D vFFR techniques which require two angiographic images and longer geometry reconstruction times. We would, however, like to challenge the authors’ claim that “computer models are complex, often time-consuming, and universally rely on the analysis of three-dimensional images that require extra acquisition, analytical, and computational steps.” The original vFFR technique [2] did require two angiographic images, and the time required to obtain the vFFR results was significantly longer than reported in the current study; however, we have demonstrated the applicability of these techniques in real-time clinical practice within the cardiac catheterisation laboratory [3]. Furthermore, simulation times have now been reduced by orders of magnitude by leveraging a graphic processing unit (GPU) computation [4].

In addition, an alternative model of flow, also requiring only a single angiographic view, has already been described [5]. This alternative single-view model invokes Murray’s law of vascular scaling [6] to regionalise side-branch flow (Q) to bifurcation points according to the vessel taper, as follows:

D α Q^c^

where D denotes vessel diameter and C is a scaling exponent. The value of C originally reported by Murray was 3.0. The validation of this single-view technique with invasive FFR measurements [5] showed similar accuracy to that reported by Tsigkas et al. [1]. This may also be improved, given the theoretical [7] and observational [8] work suggesting that the true value of the C exponent is likely within the range of 2.33–2.39 for coronary arteries. Additionally, further models have also reduced simulation time by leveraging these morphometric scaling laws to regionalise flow throughout the coronary epicardial tree from a single vessel reconstruction [9,10]. In further work, the authors may wish to compare their model against these alternative models, which may strengthen both the context and applicability to wider literature. We acknowledge the importance of the contribution made by Tsigkas et al. and will follow the future developments and validation studies of their model closely.
